# Supplementary figures and images for: Risk of Insulin Resistance and Metabolic Syndrome in Women with Hyperandrogenemia: A Comparison between PCOS Phenotypes and Beyond
Source: J Clin Med. 2021 Feb 18;10(4):829. doi: 10.3390/jcm10040829 (PMC7922675; doi:10.3390/jcm10040829)

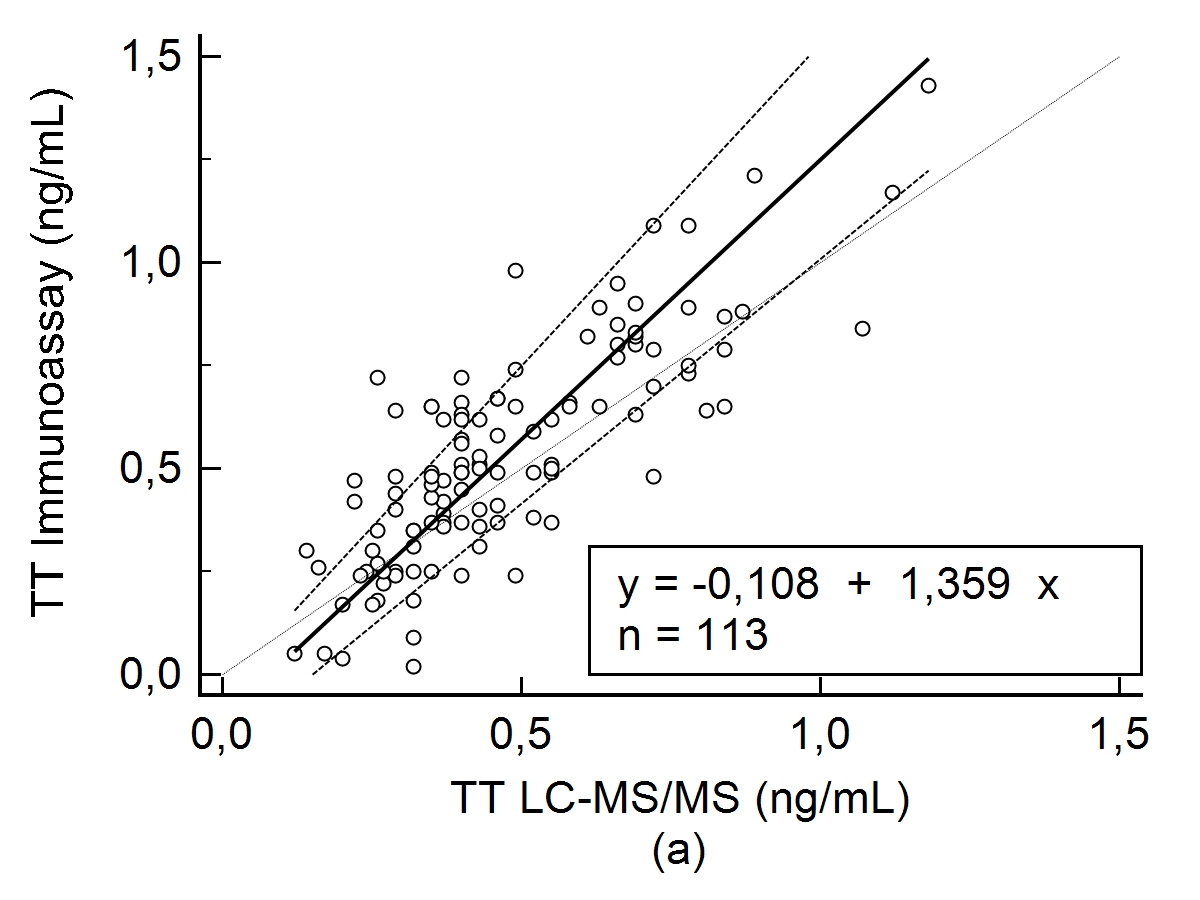

Supplement: Supplementary file 1 [file jcm-10-00829-s001.zip › Suppl. Figure 1a.jpg]

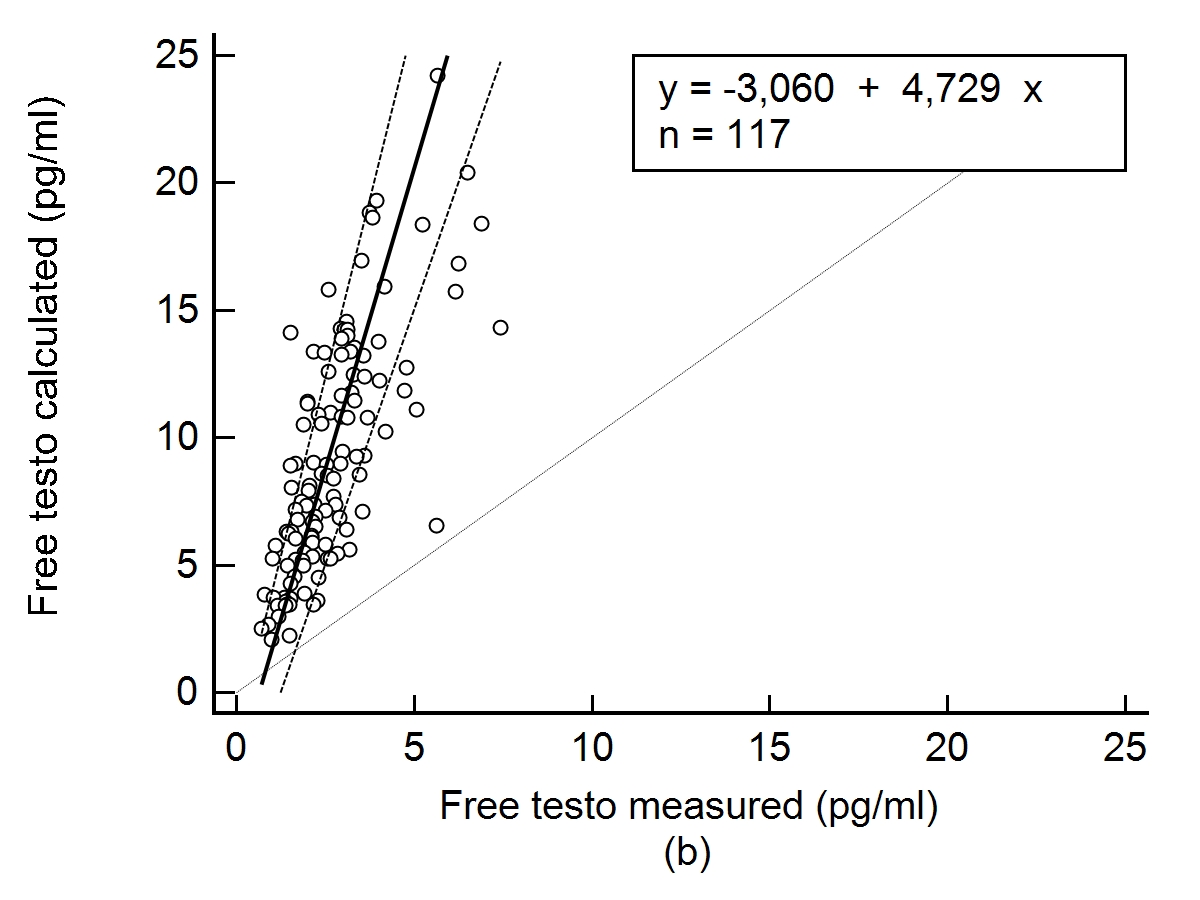

Supplement: Supplementary file 1 [file jcm-10-00829-s001.zip › Suppl_Figure 1b.jpg]
